# Phenotypic Variation of 933 Broomcorn Millet (*Panicum miliaceum* L.) Germplasm Resources

**DOI:** 10.3390/plants14162536

**Published:** 2025-08-15

**Authors:** Yuyao Kong, Xia Zhang, Haoyang Li, Yirong Qiu, Hanghang Hou, Xiaoling Zhang, Baili Feng, Qinghua Yang

**Affiliations:** 1 State Key Laboratory of Crop Stress Biology in Arid Areas, College of Agronomy, Northwest A&F University, Yangling 712000, China; kyy17838125509@163.com (Y.K.); lihy20020201@163.com (H.L.); qyr0719@163.com (Y.Q.); houhanghang@nwafu.edu.cn (H.H.); xiao01116@163.com (X.Z.); 2 Shenmu Agricultural Technology Extension Center, Shenmu 719399, China; syjs111@163.com

**Keywords:** agronomic traits, phenotypic diversity, trait association, germplasm utilisation

## Abstract

Studying comprehensive performance is fundamental for the effective utilisation of broomcorn millet (*Panicum miliaceum* L.) germplasm resources and breeding of new varieties. However, compared with other major crops, research on broomcorn millet germplasm resources is limited, and the trait variations of broomcorn millet are unclear. In this study, three qualitative and seven quantitative traits of 933 broomcorn millet core collections were analysed to provide the basis for improving utilisation of broomcorn millet germplasm resources. The seed colour was a strong phenotypic trait and had eight variants. The 933 resources exhibited three panicle types: lateral (74.5%), scattered (18.4%), and compact (7.1%). They exhibited two inflorescence colours: green (54.7%) and purple (45.3%). Pearson’s correlation analysis revealed that 1000-seed weight significantly correlated with plant height, length of panicle, and number of main stem segments. The period of duration positively correlated with 1000-seed weight but negatively correlated with the number of uniserial panicles. Cluster analysis based on the quantitative traits indicated that all resources were divided into three groups, and each group had its respective characteristics. The analysis of core germplasm resources of broomcorn millet in this study provided a basis to explore excellent genes and for breeding of excellent varieties.

## 1. Introduction

Broomcorn millet (*Panicum miliaceum* L.) is one of the ancient crops. Its cultivation can be traced back to the 6th millennium BCE in Eastern Europe and was first domesticated in China [1,2]. It has high water utilisation ability and tolerance to a broad spectrum of abiotic stresses. Therefore, it is widely cultivated in the semiarid and arid regions of Europe, Asia, and other continents [3,4]. Previous studies on broomcorn millet have mostly focused on aspects such as its genome, nutritional quality, and stress resistance, while there have been relatively few studies on its phenotypic diversity.

Studies have shown that the quality of broomcorn millet exhibits high variability, with significantly higher amylose content for the white accessions than for the coloured ones, and higher antiradical activity in the coloured accessions than in the white ones [5]. Owing to genotypic variations, certain broomcorn millet varieties demonstrate strong resistance to various abiotic stresses, especially drought and salt. This enables us to adjust crop variety selection based on local environmental conditions. In arid areas, for instance, we can choose these drought-tolerant broomcorn millet varieties, optimising crop deployment to cope with specific environmental challenges and improve overall productivity [6,7]. Broomcorn millet has rich nutritional content. It is particularly rich in flavonoids [8]. Additionally, it contains vitamins (niacin, vitamin B complex, and folic acid) and amino acids (methionine, cysteine, and isoleucine), which can make up for the nutrient deficiency of staple crops [9,10,11]. Moreover, broomcorn millet is gluten-free and suitable as an alternative diet for gluten-allergic people [12]. However, little scientific attention has been paid to broomcorn millet compared with staple crops, and the collection of its germplasm resources and related research is insufficient. This has prevented its breeding and further germplasm development. Therefore, it is very important to study the germplasm resources of broomcorn millet for its breeding and development of varieties with excellent traits.

With climate change relentlessly exerting mounting challenges to agriculture, the preservation and utilisation of broomcorn millet germplasm resources have become critical to ensure sustainable agriculture. It will offer novel solutions for mitigating agricultural problems caused by drought in the context of global warming [13]. Over 24,600 accessions of broomcorn millet collected from various regions in the world are preserved in China, Russia, and Ukraine. More than 8,500 of them are stored in the National Germplasm Resources Bank, China [14]. These abundant germplasm collections function as repositories of genetic material and hold an extensive array of valuable traits or genes. At present, the research on broomcorn millet resources mainly focuses on their nutritional components, application in soil remediation, and resistance to stresses [6,15,16,17]. However, only a few studies are available on the relationship among the traits of these resources. The agronomic traits are divided into qualitative and quantitative traits. The comprehensive evaluation of quantitative traits can provide a criterion for variety selection, and the qualitative traits can provide a clear reference for assessing the stress resistance of broomcorn millet varieties. However, the variations between qualitative and quantitative traits of broomcorn millet are unclear. In this study, we presented the phenotypic diversity of 933 broomcorn millet germplasm resources and explored the associations between their qualitative and quantitative traits, offering valuable insights for new variety breeding and the effective utilisation of existing germplasm resources.

## 2. Materials and Methods

### 2.1. Materials

A total of 933 broomcorn millet germplasm resources were kindly provided by the College of Agronomy, Northwest A&F University. Among them, 346 accessions are from Shaanxi Province, China; 21 from the Inner Mongolia Autonomous Region, China; and 18 from Heilongjiang Province, China. Additionally, 127 accessions originate from India, 64 from Turkey, and 21 from Russia, with the remaining germplasm resources coming from other regions across the globe. The research was carried out in the fields of the Miaoliang Demonstration Garden in Shenmu City, Shaanxi Province (110.51° E, 38.83° N). An experimental design method of a randomised complete block design was adopted, with three replicates being set up. All the germplasm resources were planted in sequence. Each planting area was 3 m^2^ (2 m × 1.5 m), and the row spacing was 33 cm. The spacing between plants was set at 15 cm. Approximately one month before sowing, 90 kg of urea (N ≥ 46%) per hectare was applied as the base fertiliser, and no additional fertiliser was applied thereafter. Drip irrigation was carried out both before sowing and after the seedlings emerged. For the subsequent field management measures, like weed removal and irrigation, they were implemented in accordance with the local conventional practices. All the plants were sown on 12–14 June 2023.

### 2.2. Determination of Traits

Randomly, three plants with uniform growth were selected from each plot to represent the variety. Measurements were taken, and the average values were calculated. The average values or traits from the three replicate plots were recorded as the agronomic trait indicators for this variety. During the measurement process, the main stem of the broomcorn millet was chosen. The plant height (PH) was determined as the vertical distance from the stem base to the top of the plant. The length of main panicle (LMP) was defined as the linear distance measured from the tip of the main panicle to the junction point where the panicle stalk attaches to the main stem. The length of panicle stalk (LPS) was specifically measured as the length of the stalk supporting the main panicle, from its base at the main stem to the point of panicle attachment. During the flowering stage, we used direct visual observation, and the panicle type (PT) and inflorescence colour (IC) were carefully observed. At the maturity stage, the nodes on the main stem of broomcorn millet were meticulously counted and documented as the number of main stem segments (NMSSs). Concurrently, the spikelets borne on the main panicle of broomcorn millet were carefully enumerated and designated as the number of uniserial panicles (NUPs). The sowing date and maturity date were recorded to calculate the length of the growth period (PD). After the plants reached maturity, the seeds were harvested. A seed counter was used to count out 1000 proso millet grains, which were then weighed to determine the thousand-grain weight (1000SW). After removing the outer bran, the seed colour (SC) was recorded under the same lighting conditions to ensure consistency and reproducibility of the phenotypic assessment.

### 2.3. Date Analysis

**Figure 6:**
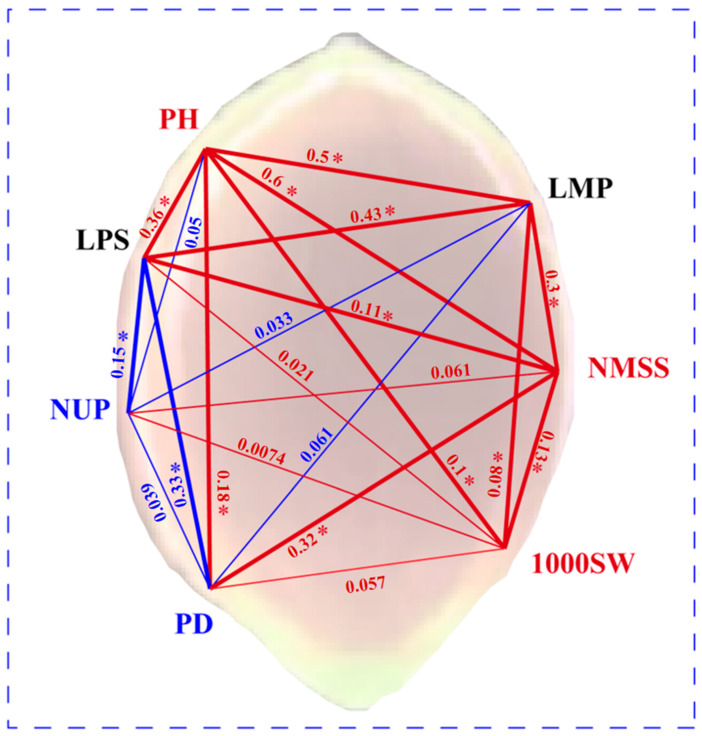
Pearson’s correlation coefficients of 7 quantitative traits. The darker the colour of the grid, the stronger the correlation. As shown by the colour bar, the deepest red corresponds to the strongest positive correlation, and the deepest blue corresponds to the strongest negative correlation. Asterisks indicate significant correlation.

Data collation and statistical analysis were carried out using Excel 2021, SPSS 27.0, and Origin 2021. Column charts and box plots were directly drawn using Origin 2021 software. For comparisons between qualitative traits and quantitative traits, pairwise statistical tests were conducted based on data distribution and variance homogeneity. Specifically, if data met the assumptions of ANOVA, Tukey’s HSD test was used for post-hoc multiple comparisons. To control Type I error caused by multiple tests, the Benjamini–Hochberg method was used for multiple testing correction, with a significance level set at *p* < 0.05. Correlation analysis was performed using Origin 2021, and a background image of broomcorn millet grains (Figure 6) was created using Adobe Illustrator 2020. Principal component analysis of quantitative traits was conducted using SPSS, and then scatter plots were drawn using Origin 2021. Cluster analysis was carried out using TBtools-II.

## 3. Results and Discussion

### 3.1. Diversity of Seed Colour

**Figure 1:**
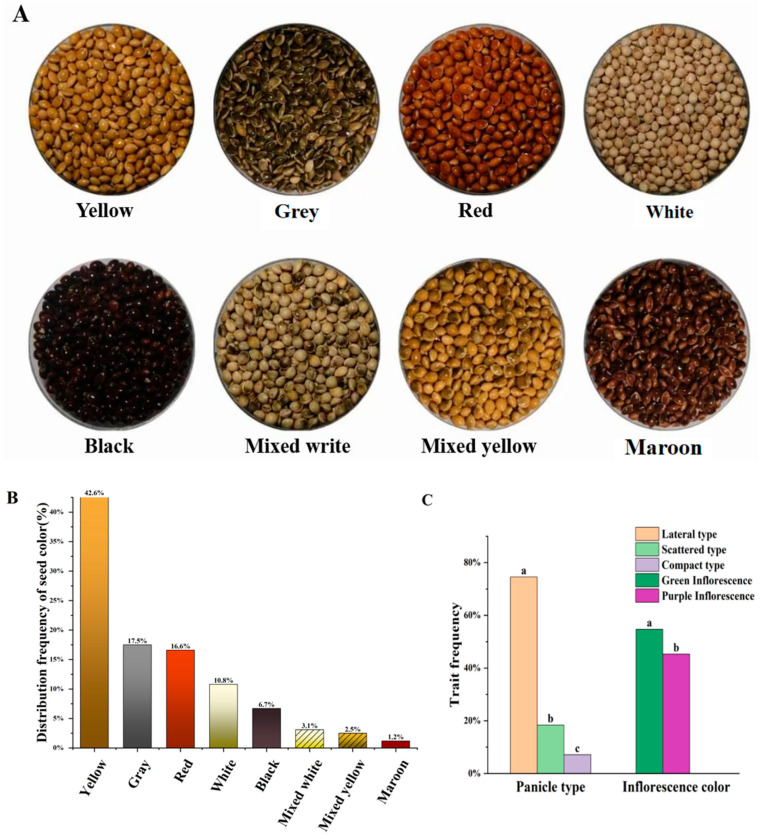
(**A**) Diverse seed colours of broomcorn millet germplasm resources. From left to right, the first line is yellow, grey, red, and white; the second line is black, mixed white, mixed yellow, and maroon. (**B**) Distribution frequency of seed colour. (**C**) Trait distribution frequency of panicle type and inflorescence colour. Panicle type: a, lateral type; b, scattered type; c, compact type. Inflorescence colour: a, green; b, purple.

Germplasm resources of broomcorn millet exhibited abundant diversity in terms of seed traits. Significant variations in colour, size, and shape were observed among various broomcorn millet varieties (Figure 1A). The 933 germplasm resources exhibited eight seed colours. Yellow seed colour was observed in the largest proportion of accessions (381; 42.6%), followed by grey (160; 17.5%) and red (152; 16.6%) seed colours (Figure 1B). The seed colour highly correlates with polyphenol content. The darker the colour of broomcorn millet seed, the higher the free phenol and free flavonoid contents [18]. These natural compounds are known for their beneficial properties, such as free radical scavenging, anticancer, and antiaging, and the effects have been proven in crops such as oats, barley and sorghum [19,20,21].

### 3.2. Diversity and Distribution of Panicle Type and Inflorescence Colour

**Figure 2:**
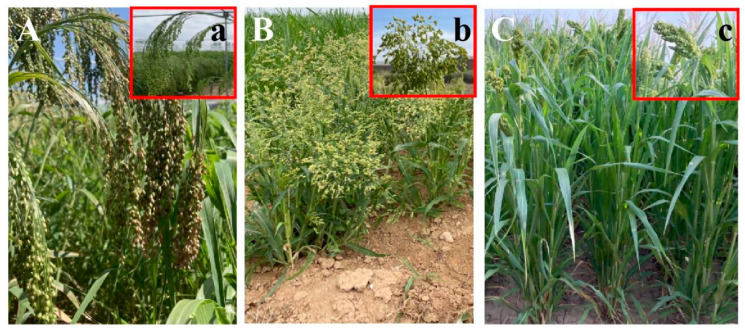
Three types of broomcorn millet panicle. (**A**,**a**), Lateral panicle type. (**B**,**b**), Scattered panicle type. (**C**,**c**), Compact panicle type.

According to the shape of the ear, broomcorn millet resources could be divided into three types (Figure 2). The lateral panicle type was observed in the highest proportion of resources (74.5%), followed by scattered panicle (18.4%) and compact panicle (7.1%) types (Figure 1C). The erect panicle type includes scattered panicle and compact panicle types and is beneficial to reduce shading under the panicle. This structure of panicle improves the photosynthetic efficiency and photosynthetic product production level of the population by comprehensively improving the population structure and light efficiency of broomcorn millet [22]. Moreover, the peduncles of broomcorn millet varieties with erect panicles are shorter, reducing the entanglement of panicles and the loss of seeds during harvesting and making them more suitable for mechanised harvesting. To promote the varieties suitable for mechanised production, the resources with the erect panicle type are important breeding materials and deserve attention, although the majority of broomcorn millet varieties in current production exhibit the lateral panicle type.

Anthocyanins regulate inflorescence colour. Their accumulation is related to glutathione s-transferase [23], which is regulated by two SNPs on chromosome 4 of broomcorn millet, resulting in green (54.7%) or purple inflorescence colours (45.3%; Figure 1C) [24]. Different inflorescence colours may occur due to long-term environmental selection. Broomcorn millet varieties with green inflorescence mainly come from low-latitude and warm areas, and those with purple inflorescence come from mostly hilly and mountainous areas with a relatively cold climate [25,26]. The variation in inflorescence colour may be an evolutionary response to attract different pollinators, thereby increasing the pollination rates and yield [27]. The underlying mechanisms of inflorescence colour dichotomy in broomcorn millet should be further explored.

### 3.3. Shannon Index of the Traits of the 933 Genotypes

The Shannon indices (H’) of 10 major traits (3 qualitative and 7 quantitative traits) of broomcorn millet germplasm resources are given in Table 1. The H’ indices of quantitative traits were significantly higher than those of qualitative traits. This is because the quantitative traits are usually characterised by continuous variation, controlled by multiple minor genes, and susceptible to environmental factors [28]. Among the qualitative traits, the highest H’ was observed for seed colour (1.641), followed by panicle type (0.718) and inflorescence (0.689). This is similar to the previous statistical results on 386 genotypes of broomcorn millet resources [29]. Among the quantitative traits, the H’ of 1000SW was the highest, followed by PH (5.421) and number of uniserial panicles (NUP; 5.473). NMSS had the lowest H’ (2.934), which was similar to the results of Zhang, et al. (2019) [29] (30.3%). The H’ of quantitative traits of the core collection of broomcorn millet resources was generally higher than that reported in a previous study [14]. This could be due to a larger number of germplasm resources in this study, which expanded the scope of quantitative traits. It may also be because the source of resources in this study was more extensive.

### 3.4. Agronomic Trait Differences of Seven Quantitative Traits Among Three Qualitative Trait Groups

#### 3.4.1. Differences in the Expression of Quantitative Traits Among Seed Colour Groups

All quantitative traits met normality assumptions (Shapiro–Wilk test, *p* > 0.05), supporting the use of one-way ANOVA. Tukey’s HSD post-hoc tests were applied, with Bonferroni correction (adjusted *p* < 0.05).

**Figure 3:**
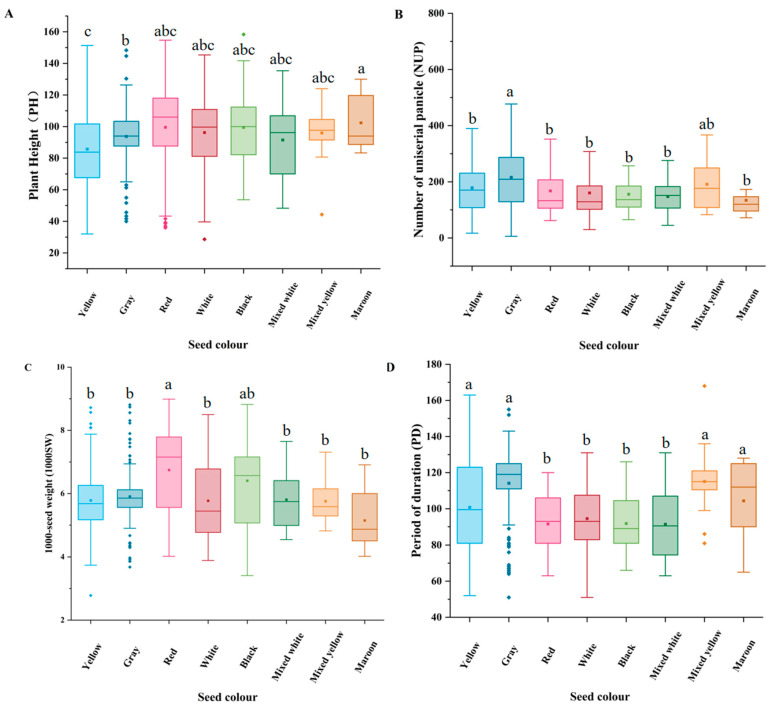
Variations of seed colour with the quantitative traits. (**A**) Plant height (PH). (**B**) Length of main panicle (LMP). (**C**) Number of main stem segment (NMSS). (**D**) Number of uniserial panicle (NUP). The small squares represent the mean, and the horizontal line in the rectangle represents the median value. Different lowercase letters (a, b, c) indicate significant differences among groups (*p* < 0.05).

The plants with maroon seed-colour genotype had the longest height (83.33–130 cm; average 102.39 cm; Figure 3A), and those with yellow seed-colour genotype had the shortest height (32–151.33 cm; average 85.71 cm). The height of plants with mixed white seed-colour genotype was 48.33–153.33 cm (average 91.49 cm). The NUP was the highest in plants with grey seed-colour genotype (average 215.8; Figure 3B), followed by those with mixed yellow, yellow, and red seed-colour genotypes. The plants with mixed white seed-colour genotype had the least NUP (147.57). Seed colour significantly correlated with the 1000SW and PD (Figure 3C,D). The plants with red seed-colour genotype had the highest 1000SW (average 6.74 g), followed by those with black seed-colour genotype (average 6.4 g). The plants with grey and mixed yellow seed-colour genotypes had the longest PD (average 114.45 and 114.06 days, respectively). The PD of plants with red seed-colour genotype was the shortest (average 91.79 days), followed by those with black (92.13 days), white (94.4 days), and mixed white (94.64 days) seed-colour genotypes.

#### 3.4.2. Differences in the Expression of Quantitative Traits Among Panicle Type Groups

**Figure 4:**
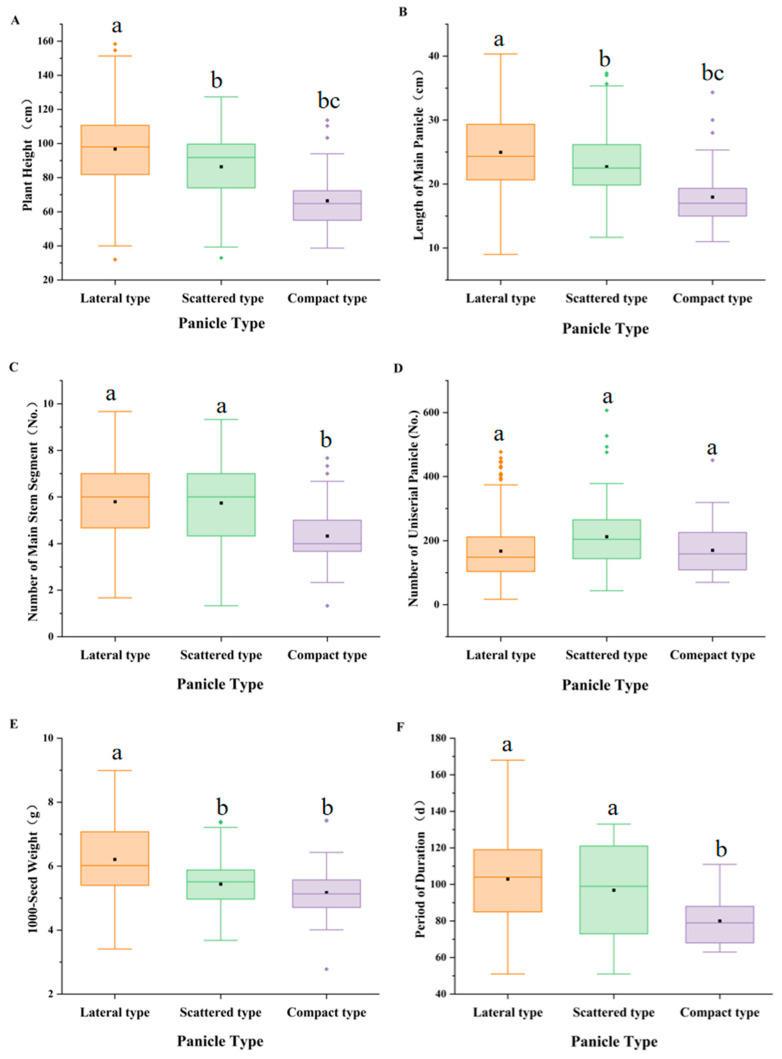
Variations of panicle type with the quantitative traits. (**A**) PH. (**B**) NUP. (**C**) 1000SW. (**D**) PD. (**E**) 1000SW. (**F**) PD. The small squares represent the mean, and the horizontal line in the rectangle represents the median value. Different lowercase letters (a, b, c) indicate significant differences among groups (*p* < 0.05).

The height of plants with the lateral panicle type was higher than that of plants with the scattered and compact panicle types, with the latter having the shortest PH (average 66.88 cm, shorter than that of the lateral type by 29.85 cm; Figure 4A). The length of main panicle (LMP) of the compact panicle type (average 17.96 cm) was approximately 39% shorter than that of the lateral panicle type (average 24.97 cm; Figure 4B). The plants with compact panicle type were shorter and more resistant to lodging. The NMSS of lateral and scattered panicle types was almost the same (average 5.79 and 5.7, respectively), whereas that of the compact panicle type was the least (average 4.32; Figure 4C). The panicle type affected the lodging resistance through the bending movement of the stem. The bending movement of the compact panicle type was less; therefore, the centre of gravity was relatively low, and the lodging resistance was strong [30]. The plants with scattered panicle type had the maximum NUP (up to 212.72 on average; Figure 4D), and those with lateral panicle type had the highest 1000SW (6.21 g; Figure 4E). The PD of plants with lateral panicle type was the longest (average 102.96%; 28.5% longer than that of plants with the compact panicle type; Figure 4F). This variability enables crops to acclimatise to changing environmental patterns [31].

#### 3.4.3. Differences in the Expression of Quantitative Traits Among Inflorescence Colour Groups

**Figure 5:**
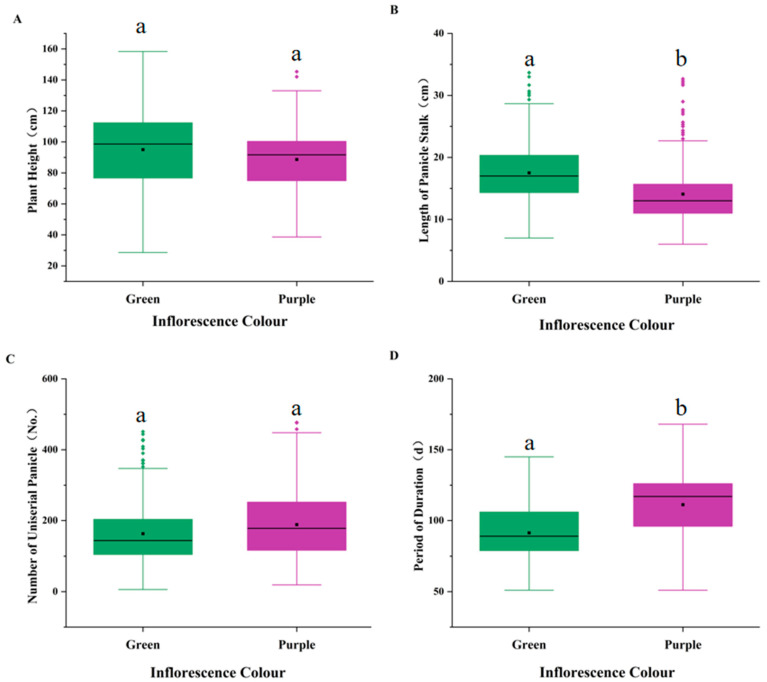
Variations of inflorescence colour with quantitative traits. (**A**) PH. (**B**) LPS. (**C**) NUP. (**D**) PD. The small squares represent the mean, and the horizontal line in the rectangle represents the median value. Different lowercase letters (a, b, c) indicate significant differences among groups (*p* < 0.05).

PH is a crucial trait affecting lodging resistance and yield of broomcorn millet [32]. The plants with green inflorescence had a higher PH (average 95.01 cm; 6.2 cm higher than those with purple inflorescence; Figure 5A). The length of panicle stalk (LPS) of plants with green inflorescence was 23.5% higher than that of plants with purple inflorescence (Figure 5B). However, the plants with purple inflorescence had better lodging resistance because of lower PH and shorter panicle stalk. The number of panicles in the plants with purple inflorescence was 188.58 on average, 15.67% higher than that in the plants with green germplasms (Figure 5C). The yield of plants with purple inflorescence was higher than that of plants with green inflorescence. This can be attributed to two reasons. First, decreased stem growth rate during panicle development resulted in more fertile florets and more seeds per m^2^ in plants with purple inflorescence. Second, their fertiliser utilisation rate was higher because they were less susceptible to lodging [33]. Because of the natural selection and genetic variation, the PD of plants with purple inflorescence (111.23 days) was significantly longer than that of plants with green inflorescence (91.34 days; Figure 5D).

### 3.5. Pearson’s Correlation Analysis for the Quantitative Traits

Pearson’s correlation analysis was conducted to analyse the interactions among the quantitative traits (Figure 6). The PH correlated with LMP, LPS, NMSS, 1000SW, and PD (*p* < 0.05). This indicated that both the length of the panicle and stem of the plant increased with the PH, which requires more time for growth. Major quantitative trait loci in chromosomes that affect PH and other quantitative traits should be mapped in future studies because they will lay a foundation for further understanding the molecular mechanism of regulation of quantitative traits in broomcorn millet [32]. The 1000SW significantly correlated with PH, LMP, and NMSS and has a non-significant positive correlation with PD. Appropriate PH, stalk length, and excellent lodging resistance are the key factors responsible for the final yield of broomcorn millet; therefore, further study to assess the genes responsible for these traits is necessary. LPS was significantly negatively correlated with NUP and PD (*p* < 0.05).

### 3.6. Principal Component Analysis

**Figure 7:**
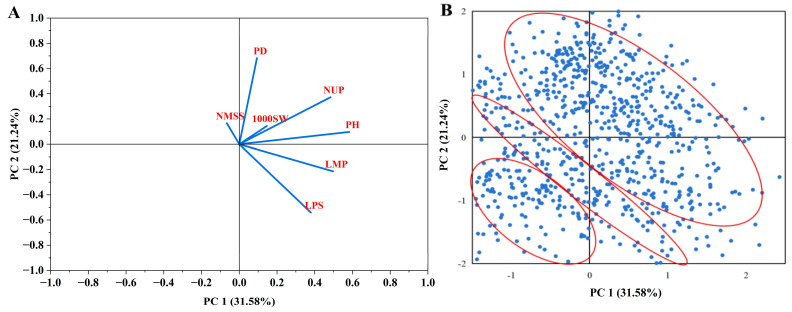
Principal component analysis (PCA). (**A**) PCA of all traits. The blue lines represent the loads. (**B**) PCA of 933 genotypes for PH, LMP, NMSS, NUP, 1000SW, and PD. The three red ellipses represent confidence ellipses with a confidence interval of 95%.

To reduce and visualise the data for quantitative traits, principal component analysis was performed. The cumulative contributions to the total variation for the first to third principal components reached >67.53%, which was sufficient to represent a large part of the original indicator information. Therefore, three independent comprehensive indicators could be adapted to objectively analyse the quantitative trait characteristics of the 933 germplasm resources. The 933 broomcorn millet germplasm resources were roughly divided into three parts (Figure 7B), and the contribution of three quantitative traits to the grouping is shown in Figure 7A.

The first principal component had the largest contribution rate and eigenvalue (31.68% and 2.22, respectively) (Table 2). In the first principal component feature vector, PH, LMP, NMSS, and LPS had higher load and positive values, and their feature vector values were 0.867, 0.743, 0.720, and 0.565, respectively. Such agronomic traits were related to the PH of broomcorn millet. The agronomic trait with high load and negative value was NUP, which is closely related to yield. Similarly, the eigenvalue of the second principal component was 1.49, and the contribution rate was 21.29%. The agronomic trait with the highest load and positive value was PD, and the feature vector value was 0.832. If the growth period is too short, the seed formation will be affected. If the growth period is too long, it will cause “greedy green” (plant maintaining high chlorophyll content for an extended time even after maturation) and late ripening. Therefore, broomcorn millet varieties with different PD are needed for introduction, cultivation management, and variety layout, and the crop yields can be increased by timely adaptation of growing periods to climate change [34]. The agronomic trait with the highest load and negative value was LPS, and its eigenvector value was −0.66. If the peduncle is too long, its pressure would lead to plant lodging. The contribution rate of the third principal component was 14.57%. In terms of the magnitude of the eigenvalues, the third principal component mainly reflected the LMP, NUP, and 1000SW and could be defined as a yield factor.

### 3.7. Cluster Analysis of the Germplasm Resources Based on the Quantitative Traits

**Figure 8:**
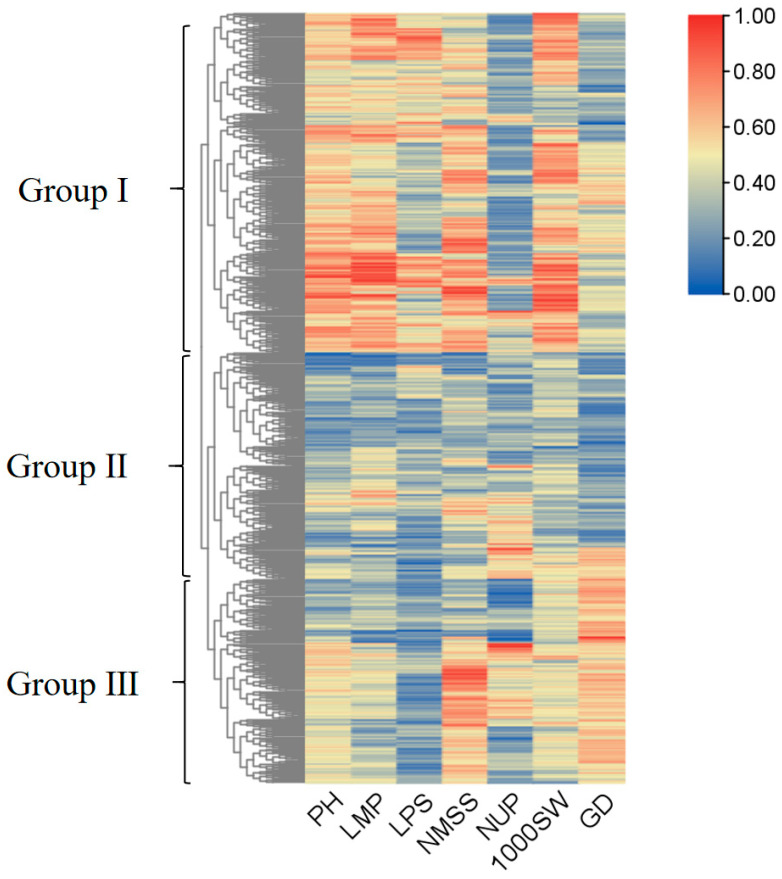
Clustering of broomcorn millet varieties based on 7 quantitative traits and the Complete (complete linkage method) was used.

Based on the seven quantitative traits, 933 broomcorn millet varieties were grouped into three categories by cluster analysis (Groups I, II, and III; Figure 8). This suggested that each cultivated population developed different phenotypes to adapt to the local environment. In Group I, most broomcorn millet varieties had larger PH, LMP, NMSS, and 1000SW. In Group II, all agronomic traits were at a low level, and the PD of almost all varieties was short. A shorter growth period can lead to a shorter time required for crop reproduction and earlier harvest [35]. Short-growth-period crops enhance agricultural resilience through multiple pathways. Rapid rotation maximises land use efficiency, enabling 2–3 annual harvests. Post-disaster reseeding (e.g., buckwheat) ensures rapid yield recovery, mitigating food crises. Studies confirm these varieties reduce heavy metal uptake (e.g., Cd/As) via phytoextraction, aiding contaminated soil remediation. [36,37]. The remaining germplasm resources were assigned to Group III, which had higher NMSS and PD and shorter LPS. However, the germplasm resources with a longer growth period are suitable for early sowing in the semiarid area [38]. The longer growth period means that the nutrients in the seed of broomcorn millet accumulate over a longer period of time. Therefore, the varieties with high nutritional quality can be selected from this group [39].

## 4. Conclusions

The 933 broomcorn millet resources had rich phenotypic diversity, and variations were observed among different traits. The broomcorn millet resources with purple inflorescence, compact panicle type, and yellow seeds had shorter height and higher lodging resistance. Genes related to the dwarf trait can be mined from them. The germplasm resources with lateral panicle type and red or black seeds had larger 1000SW; those with purple inflorescence, scattered panicle type, and grey seeds had more NUP. These varieties should be selected for high-yield breeding. The germplasm resources with green inflorescence, compact panicle type, and red seeds had a shorter growth period and are suitable for reseeding to restore agricultural production. Furthermore, the germplasm resources with outstanding useful characteristics were screened; they can be used for the selection of parents for breeding improvement. These 933 germplasm resources have abundant traits and can be used for the evaluation of dominant traits and localisation of dominant genes in the future.

## Figures and Tables

**Table 1:** The Shannon index (H′) of broomcorn millet germplasm resources.

Trait	H′	Trait	H′
Seed colour (SC)	1.641	Length of panicle stalk (LPS)	4.034
Inflorescence colour (IC)	0.689	Number of main stem segment (NMSS)	2.934
Panicle type (PT)	0.718	Number of uniserial panicle (NUP)	5.473
Plant height (PH)	5.421	1000-seed weight (1000SW)	6.358
Length of main panicle (LMP)	4.261	Period of duration (PD)	4.253

**Table 2:** Power vector (PV), eigenvalues (E), contribution rate (CR), and cumulative contribution rate (CCR) of the first 4 principal components based on the 7 phenotypic traits. Note: PH, plant height; LMP, length of main panicle; LPS, length of panicle stalk; NMSS, number of main stem segments; NUP, Number of uniserial panicle; 1000SW, 1000-seed weight; PD, Period of duration. Principal component analysis was stopped when the cumulative contribution rate was greater than 80%.

Trait ^(1)^	PV (1)	PV (2)	PV (3)	PV (4)
PH	0.867	0.122	−0.24	−0.114
LMP	0.743	−0.266	0.092	−0.056
LPS	0.565	−0.660	−0.023	0.014
NMSS	0.720	0.453	0.067	−0.113
NUP	−0.92	0.200	0.938	−0.214
1000SW	0.219	0.177	0.202	0.938
PD	0.142	0.832	−0.290	−0.077
E	2.218	1.490	1.020	0.960
CR (%)	31.680	21.287	14.567	13.717
CCR (%)	31.680	52.967	67.534	81.252

## Data Availability

All data generated or analysed during this study are included in this published article.

## Abbreviations

SC, seed colour; LPS, length of panicle stalk; IC, inflorescence colour; NMSS, number of main stem segment; PT, panicle type; NUP, number of uniserial panicle; PH, plant height; 1000SW, 1000-seed weight; LMP, length of main panicle; PD, period of duration.
